# Long-term outcomes of arthroscopic Bankart repair: a 10-year follow-up study

**DOI:** 10.1016/j.jseint.2025.07.004

**Published:** 2025-08-05

**Authors:** Shogo Sugawara, Nobuyuki Yamamoto, Yusuke Koibuchi, Kazuma Sasaki, Rei Kimura, Atsushi Arino, Jun Kawakami, Hideaki Nagamoto, Toshimi Aizawa, Eiji Itoi

**Affiliations:** a Department of Orthopaedic Surgery, Kurihara City Central Hospital, Kurihara, Japan; b Department of Orthopaedic Surgery, Tohoku University School of Medicine, Sendai, Japan; c Department of Orthopaedic Surgery, South Miyagi Medical Center, Ogawara, Japan; d Graduate School of Sport Science, Waseda University, Tokorozawa, Japan; e Department of Orthopaedic Surgery, Tohoku Rosai Hospital, Sendai, Japan

**Keywords:** Arthroscopic Bankart repair, Anterior shoulder dislocation, Long-term outcome, Osteoarthritis, Subluxation, Instability

## Abstract

**Background:**

Arthroscopic Bankart repair (ABR) is the gold standard for anterior shoulder dislocation, with many studies demonstrating favorable short-term outcomes. However, few reports focus on the long-term outcomes, partly due to a challenge of following up younger patients. This study aims to evaluate the long-term outcomes of ABR.

**Methods:**

This study included 32 shoulders (19 males, 13 females; mean age at surgery: 31 years) out of 55 shoulders that underwent ABR at our institution between 2011 and 2019. Inclusion criteria required a minimum follow-up period of 5 years, and data were collected through telephone surveys or direct examinations. The survey assessed recurrent dislocation or subluxation, shoulder pain, apprehension, return to sports (complete, partial, or inability), and revision surgery. Direct examinations included physical assessments and plain x-rays. The Rowe score and Western Ontario Shoulder Instability Index score were assessed. Adjunct data on surgical procedures and intra-articular lesions were also reviewed.

**Results:**

The mean follow-up period was 10 years (range: 6-14 years). Recurrent dislocation occurred in 3 shoulders (10%), and subluxation in 7 shoulders (22%), yielding a total recurrence rate of 28%. Recurrences were associated with sports or traumatic events. Sports return rates were 72% (23 shoulders) for complete return, 16% (5 shoulders) for partial return, and 13% (4 shoulders) for inability (rugby, volleyball, and judo). Shoulder pain (2-3 in numeric rating scale) was reported in 2 shoulders, and apprehension in the abduction and external rotation position was observed in 4 shoulders (13%). The Rowe score significantly improved from 44 ± 8 points (mean ± standard deviation) to 87 ± 21 points (*P* < .05), and no revision surgeries were performed. Mild to moderate osteoarthritis on X-rays were found in 2 shoulders.

**Conclusion:**

The average 10-year postoperative recurrence rate was 28%, consistent with the previous systematic reviews. While the recurrence rates increased over time, patient satisfaction remained high, and no revision surgeries were required. These findings demonstrate the long-term reliability of ABR for anterior shoulder instability.

Arthroscopic Bankart repair (ABR) is the gold standard for recurrent anterior shoulder dislocation. However, a high rate of recurrence has been reported in patients with large glenoid bony defects, Hill–Sachs lesions, and those engaged in contact or collision sports.^2^^,^^24^^,^^25^ In such high-risk cases, coracoid transfer procedures have been increasingly performed.^29^^,^^33^ Nevertheless, the risk of recurrence remains substantial. Several studies have suggested that adjunct procedures, such as rotator interval closure or remplissage, in addition to ABR, may improve outcomes in these high-risk cases.^6^^,^^8^^,^^25^^,^^37^

Most studies evaluating ABR have focused on short-term outcomes. There are not many studies reporting with a follow-up of more than five years after ABR. The available long-term studies suggest that the recurrence rate of dislocation increases over time, ranging from 13% in the short-term to 31% in the long-term.^10^^,^^21^ Therefore, the purpose of this study was to evaluate the mid- to long-term outcomes of patients who underwent ABR with a minimum follow-up of five years. We hypothesized that mid- to long-term outcomes would be inferior to short-term results.

## Subjects and methods

The subjects were 55 shoulders that underwent ABR for traumatic recurrent anterior shoulder dislocation or subluxation between 2011 and 2019. Of these, 32 patients who met the following inclusion criteria were retrospectively reviewed: (1) those who underwent ABR with and without adjunct procedures; (2) those who were eligible for either a telephone survey or direct examination; and (3) a minimum follow-up of 5 years. Exclusion criteria were as follows: (1) patients with a glenoid defect of greater than 25% of the glenoid width,^12^; (2) patients with an off-track Hill–Sachs lesion^38^; (3) revision Bankart repairs; (4) patients with full-thickness rotator cuff tears; and (5) patients with a first-time dislocation. All patients underwent preoperative computed tomography (CT) and magnetic resonance imaging examinations. Glenoid bone loss was measured using CT, including 3-dimensional CT, and the presence of soft tissue injuries, such as Bankart lesions and rotator cuff tears was assessed with magnetic resonance imaging. Patients were selected according to the exclusion criteria based on these imaging findings. Six patients met the exclusion criteria. First, all patients were contacted and verbally surveyed to assess the presence of recurrent dislocation or subluxation, shoulder pain, apprehensive feeling during daily activities and sports, return to sports (categorized as full, partial, inability, or quit), and any history of revision surgery. Patients who were able to visit the institute underwent a comprehensive physical examination, including an assessment of shoulder range of motion, muscle strength, and the anterior apprehension test. In addition, plain X-rays were obtained. Clinical outcomes were evaluated using the Rowe score,^31^ a physician-led clinical evaluation method, while patient-reported outcomes were assessed with the Western Ontario Shoulder Instability Index (WOSI).^16^ Pain severity was quantified using the Numerical Rating Scale. X-ray findings were evaluated according to the Samilson and Prieto classification.^32^ Furthermore, medical records and images were reviewed to determine the presence of arthropathy, Hill–Sachs lesions (categorized as on- or off-track lesions), and other intra-articular lesions. The surgical procedures were assessed for the inclusion of additional interventions such as rotator interval closure, remplissage, or capsular plication, in conjunction with Bankart repair. This study was approved by the institutional review board (# 2013-1-321). A paired *t*-test was performed using a statistical software (EZR; Saitama Medical Center, Jichi Medical University, Saitama, Japan) to compare the preoperative and postoperative Rowe and WOSI scores, with a significance level set at 0.05.

### Surgical procedure

All operations were performed with the patient under general anesthesia by 2 senior surgeons (EI and NY). ABR was performed with the patient in the beach-chair position. Two portals were established—antero-superior and anteroinferior portals—in addition to the standard posterior viewing portal. The inferior glenohumeral ligament-labrum complex was mobilized with use of an elevator. The anterior neck of the glenoid was roughened by the rasp. Bioabsorbable suture anchors (Gryphon BR; DePuy Mitek, Raynham, MA, USA) were used with a minimum of 3 anchors (mean: 3.8). Sutures were passed through the detached labrum with use of a suture passer (Accu-Pass; Smith & Nephew, Andover, MA, USA). The arthroscopic technique included a routine incorporation of capsular plication and proximal shift. When there was anterior laxity of the glenohumeral joint under anesthesia compared with the contralateral side (4 of 32 shoulders), the rotator interval closure was added with FiberWire sutures (Arthrex, Inc., Naples, FL, USA). Postoperative rehabilitation consisted of external immobilization in internal rotation using a brace for three weeks. Starting three weeks after surgery, patients underwent unrestricted range-of-motion and scapular stabilization exercises on an outpatient basis for six months.

## Results

The results are summarized in Table I. The postoperative follow-up period ranged from 6 to 14 years, with an average 9.8 years. The mean age at the time of surgery was 31 years. The number of preoperative dislocation episodes ranged from 2 to 30, with an average of 6.2. Additional procedures during Bankart repair were rotator interval closure in 4 shoulders and capsular plication in 9 shoulders. No patient underwent remplissage. A telephone survey revealed that 3 shoulders (9%) had recurrent dislocation and 7 shoulders (22%) subluxation, resulting in an overall recurrence rate of 28% (9 shoulders). All recurrent dislocations occurred within the first postoperative year due to trauma. Among the 7 cases of subluxation, 5 occurred within the first year, 1 occurred 10 years postoperatively, and 1 followed a fall at an unknown time. None of the patients required revision surgery.

**Table I:** Demographic and clinical characteristics of patients who underwent arthroscopic Bankart repair.

	Age at surgery (yr)	Preoperative dislocation episodes	Follow-up period (yr)	Sex	Preoperative sports	Preoperative		Adjunct procedures			Postoperative				
						Glenoid defect (%)	Off-track Hill–Sachs lesion	Rotator interval closure	Capsular plication	Remplissage	Dislocation	Subluxation	Pain	Return to sports	Rowe score
Case 1	49	30<	14	F	Swimming	13.5	-	-	+	-	-	-	-	Partial	100
Case 2	21	3	13	M	Baseball	11.8	-	-	-	-	-	-	-	Full	100
Case 3	31	5	13	M	Golf	9.3	-	-	-	-	-	-	-	Full	95
Case 4	43	4	11	M	Judo	19.5	-	-	-	-	-	-	-	Impossible	95
Case 5	35	20<	12	M	-	2.3	-	-	-	-	-	-	-	Full	100
Case 6	57	3	12	F	Golf	1.1	-	-	-	-	-	-	+	Full	100
Case 7	53	10	12	F	Swimming	2.4	-	-	-	-	-	+	-	Full	60
Case 8	20	6	12	M	-	19.6	-	-	-	-	-	-	-	Full	80
Case 9	19	3	12	M	Volleyball	1.1	-	-	-	-	+	-	-	Impossible	45
Case 10	66	6	12	F	-	16.7	-	-	-	-	-	+	-	Full	60
Case 11	25	4	11	M	Soccer	12.5	-	-	-	-	+	+	-	Partial	45
Case 12	18	3	10	F	Volleyball	6.6	-	-	+	-	-	-	-	Full	100
Case 13	47	5	11	F	-	8.7	-	-	-	-	-	-	+	Full	100
Case 14	17	10<	12	M	-	21.7	-	-	-	-	-	-	-	Full	100
Case 15	20	4<	10	M	-	12.6	-	-	+	-	-	-	-	Full	100
Case 16	36	10	8	M	-	6.1	-	-	-	-	-	-	-	Full	75
Case 17	27	10<	11	M	Judo	18.7	-	-	+	-	-	-	-	Full	75
Case 18	23	3	9	M	-	1.1	-	-	+	-	-	-	-	Partial	55
Case 19	31	7	9	F	-	12.9	-	-	+	-	-	-	-	Full	100
Case 20	19	2	8	M	Rugby	3.5	-	-	-	-	-	+	-	Impossible	50
Case 21	42	3	8	M	Golf	4.6	-	+	+	-	-	-	-	Full	95
Case 22	21	6	9	M	Athletics	10.1	-	-	-	-	-	+	-	Full	60
Case 23	20	3	8	M	-	11.9	-	-	-	-	-	+	-	Impossible	55
Case 24	44	4	9	M	-	7.2	-	-	-	-	-	-	-	Full	75
Case 25	43	5	8	M	-	2.5	-	-	-	-	-	-	-	Full	80
Case 26	20	4	6	F	Judo	7.1	-	-	-	-	-	-	-	Full	95
Case 27	17	3	7	F	-	15.9	-	+	+	-	-	+	-	Partial	60
Case 28	21	6	7	F	-	2.6	-	+	-	-	-	-	-	Full	100
Case 29	26	3	6	M	Soccer	9.8	-	-	-	-	+	-	-	Partial	45
Case 30	19	4	8	F	Badminton	4.9	-	+	+	-	-	-	-	Full	100
Case 31	17	3	6	M	Combat sport	12.4	-	-	-	-	-	-	-	Full	100
Case 32	35	5	10	F	-	5.1	-	-	-	-	-	-	-	Full	100
Mean	30.7	6.2	9.8			9.2									81.3

Four patients (13%) reported apprehension in the abducted and external rotation (ER) position, and 2 patients (6.3%) (a 47-year-old female and a 57-year-old female) had residual pain, rated 2-3 on the Numerical Rating Scale. All 32 patients had preoperative Rowe scores. Mean preoperative Rowe score of 44.6 ± 7.8 significantly improved to 81.3 ± 20.6 postoperatively (*P* < .05). Seventeen patients participated in daily sports activities preoperatively. Postoperatively, 4 patients resumed daily sports participation, while 11 returned to recreational-level sports. Overall, 23 patients (72%) returned to sports fully, 5 (16%) returned with some restrictions, and 4 (13%) were unable to resume sports. The sports in which they were unable to return included judo, volleyball, and rugby.

Verbal reports of daily activity limitations included decreased range of motion in 5 patients (16%), apprehensive feeling in 4 patients (13%), and muscle weakness in 3 (10%).

**Figure 1:**
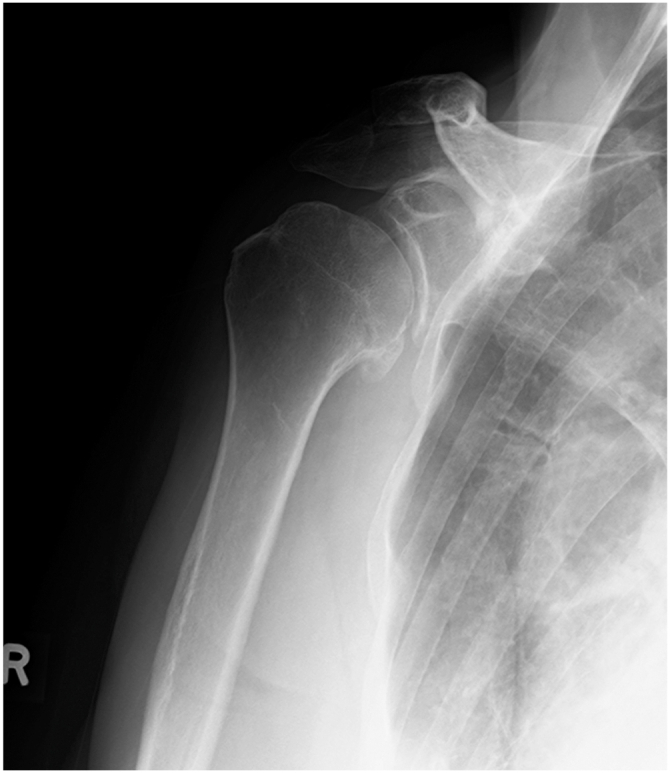
X-ray of a 64-year-old female 14 years after surgery. This X-ray showed moderate osteoarthritic changes.

Physical examination was performed in 7 patients (Table II). The mean postoperative range of motion for flexion was 173 ± 6° on the unaffected side and 171 ± 8° on the affected side. ER measured 78 ± 16° on the healthy side and 76 ± 19° on the affected side. One shoulder exhibited a positive apprehension test, and the WOSI score averaged 89%. Plain X-rays revealed osteoarthritic changes in 2 shoulders, classified as mild and moderate according to the Samilson and Prieto classification. These 2 patients were in their 40s (Fig. 1).

**Table II:** Data of cases with physical examination.

Case	Range of shoulder motion (degrees)										Anterior apprehension test	Muscle strength		X-ray†
	Involved side					Uninvolved side								
	Elv	ER	IR∗	ER90	IR90	Elv	ER	IR∗	ER90	IR90		Supraspinatus	Infraspinatous	
1	170	80	T8	80	80	170	80	T8	80	80	-	Normal	Normal	OA class Ⅱ
3	180	90	T5	90	80	180	90	T5	90	80	-	Normal	Normal	-
6	160	45	T12	90	70	160	45	T12	90	70	-	Normal	Normal	-
19	170	90	T8	90	80	170	90	T8	90	80	-	Normal	Normal	-
22	180	90	T10	100	90	180	90	T10	110	90	+	Normal	Normal	-
25	160	45	T8	80	60	170	60	T8	90	70	-	Normal	Normal	OA class Ⅰ
28	180	90	T8	90	80	180	90	T8	90	80	-	Normal	Normal	-

*Elv*, elevation; *ER*, external rotation; *IR*, internal rotation; *ER90*, external rotation in abduction; *IR90*, internal rotation in abduction; *OA*, osteoarthritis; *T*, thoracic vertebral level.

∗ Vertebral level that can be reached behind the back.

† Samilson and Pietro classification.

## Discussions

In this study, telephone surveys were conducted for 25 patients, while 7 patients underwent direct clinical examination, resulting in a follow-up rate of 32 out of 55 shoulders (58%). The mean postoperative follow-up period was 10 years. Recurrent dislocation occurred in 3 shoulders (10%) and subluxation in 7 (22%), leading to an overall recurrence rate of 28% (9 shoulders). No patients required revision surgery. All instances of recurrent instability (dislocation or subluxation) were associated with sports activities or trauma, such as falls. Regarding return to sports, 23 patients (72%) resumed full participation, 5 patients (16%) returned with some restrictions, and 4 (13%) unable to return. Osteoarthritic progression was observed in 2 of the 7 shoulders that underwent X-ray examination. These 2 patients were 49 and 43 years of age.

In the short term, the recurrence rate of dislocation following ABR has been reported to range from 5% to 10%.^1^^,^^7^^,^^9^^,^^15^^,^^17^^,^^19^^,^^20^^,^^25^^,^^26^,^29^^,^^30^^,^^35^^,^^36^ However, studies examining mid- to long-term outcomes are limited. In the mid-term follow-up, Carriere et al^4^ reported a 10% recurrence rate at 4 years, Kandziora et al^13^ found a 16% recurrence rate at 4.5 years, and Vermeulen et al^34^ observed a 22% recurrence rate at 6.3 years, indicating a trend of increase compared to short-term results. Regarding the long-term outcomes, Murphy et al^21^ reported a 31% recurrence rate with a mean follow-up of 12 years, while Ono et al^22^ found a 31.4% recurrence rate after ABR at an average follow-up of 10 years. Castagna et al^5^ reported a 16% recurrence rate at 10 years. These studies collectively demonstrate a progressive increase in recurrence rate over time compared to mid-term outcomes.

Several studies have investigated the WOSI score as patient-reported outcome measures. Kavaja et al^14^ reported the WOSI score of 78% at the final follow-up after 13 years, while Privitera et al^28^ and Owens et al^23^ reported 83% at 13.5 years and 82% at 11 years, respectively, indicating a long-term satisfaction rate of approximately 80%. In the present study, the mean WOSI score at the final follow-up was 89%, comparable to or higher than previously reported. These findings suggest that although the recurrence rate of instability, including subluxation, increased to 28% over time following ABR, patient-reported satisfaction remained high.

Regarding return to sports, Harris et al^10^ reported that 87% of patients resumed competition at the same level as before their injury after a mean follow-up of 7.3 years. Similarly, Castagna et al^5^ found that 71% of patients returned to their preoperative level of competition after 10 years. In the present study, the overall return-to-sport rate was 88% when including patients with restricted participation. Postoperative range of motion was well preserved, with ER in abduction measuring 76 ± 19° and 89 ± 6°, with differences of only 2° compared to the healthy side. Muscle strength was also maintained, which may have contributed to the high return-to-play rate. However, some patients experienced limitations or were unable to return to contact or overhead sports due to apprehensive feeling.

In the present study, cases with > 25% glenoid bone loss, off-track Hill–Sachs lesions, and prior revision surgery were excluded,^2^^,^^11^^,^^18^^,^^38^ as these were known risk factors for failure following ABR. In such cases, coracoid transfer has been our preferred approach. The findings of this study suggest that by carefully selecting surgical candidates, ABR can achieve high patient satisfaction and favorable long-term outcomes. These results seem to be useful when determining surgical indications.

Degenerative changes of the glenohumeral joint are a well-documented long-term consequence of ABR,^10^^,^^27^ with incidence rates ranging from 37% to 60%. In the present study, degenerative changes were observed in 2 of 7 examined shoulders (29%), both in their 40s. Furthermore, Buscayret et al^3^ reported that the severity of arthropathic changes correlates with increasing age at the time of initial dislocation and surgery, a trend that was also observed in this study. When performing ABR in patients in their 40s, it is necessary to explain preoperatively that there is a high risk of developing osteoarthritis in the mid- to long-term follow-ups.

This study had several limitations. First, the sample size was relatively small, particularly for cases in which physical examination was performed. While telephone interviews were feasible, many patients had moved away due to employment or job transfers, making in-person reassessment challenging. Second, the decision to perform additional augmentation procedures, such as capsular plication, was at the discretion of the surgeon. The inclusion of additional procedures may have influenced the recurrence rate. Third, there was variability in preoperative sports and activity levels. A higher proportion of contact or collision athletes, as well as overhead throwing athletes, could potentially impact postoperative outcomes. Fourth, the mean age at the time of surgery in this study was 31 years. If more patients in their teens and twenties had been included and the average age had been lower, the recurrence rate observed in this study might have been higher. Future, large-scale studies are required to further investigate these factors.

## Conclusion

The average 10-year postoperative recurrence rate was 28%, consistent with the previous systematic reviews. While the recurrence rate of dislocation increased over an average follow-up of 10 years after surgery, patient satisfaction remained high, and no revision surgeries were required. These findings demonstrate the long-term reliability of ABR for anterior shoulder instability.

## Disclaimers

Funding: No funding was disclosed by the authors.

Conflicts of interest: The authors, their immediate families, and any research foundation with which they are affiliated have not received any financial payments or other benefits from any commercial entity related to the subject of this article.
